# Host cell protein impurities modify the immunomodulatory profile of pregnancy-specific glycoprotein 9, a cautionary tale

**DOI:** 10.3389/fimmu.2026.1882381

**Published:** 2026-10-14

**Authors:** Mirian Mendoza, Uriel Ortega-Rodriguez, Ha-Na Lee, Angela Ballesteros, James Warren, Maria Rendon-Correa, Wells W. Wu, Daniela Verthelyi, Gabriela Dveksler

**Affiliations:** 1 Office of Pharmaceutical Quality Research, Office of Pharmaceutical Quality, Center for Drug Evaluation and Research, U.S. Food and Drug Administration, Silver Spring, MD, United States; 2 National Institute on Deafness and Other Communication Disorders (NIH), Section on Sensory Physiology and Biophysics, Bethesda, MD, United States; 3 Uniformed Services University of the Health Sciences, Pathology, Bethesda, MD, United States; 4 Facility for Biotechnology Resources, Center for Biologics Evaluation and Research, Food and Drug Administration, Silver Spring, MD, United States

**Keywords:** host cell protein, immunomodulatory, impurity, product quality, recombinant pregnancy-specific glycoproteins

## Abstract

**Introduction:**

Pregnancy-specific glycoprotein 9 (PSG9) is one of 10 members of a conserved glycoprotein family within the carcinoembryonic antigen superfamily expressed by placental trophoblasts and secreted into maternal circulation. PSG1 and PSG9 induce the conversion of naïve T cells into regulatory T cells by increasing active transforming growth factor-beta (TGF-β) availability and were predicted to share anti-inflammatory activity in innate immune cells. However, while maternal PSG1 concentrations are lower in preeclampsia, PSG9 levels are reported to be elevated, suggesting PSG1 and PSG9 may differ in some immune-regulatory functions. Here, we characterized the immunomodulatory effects of PSG9 on myeloid cells.

**Methods:**

Recombinant PSG9 was produced in Chinese hamster ovary (CHO) cells using standard expression protocols and evaluated for biological activity in human and murine myeloid cells.

**Results:**

Results: Recombinant PSG9 preparations initially displayed batch-dependent variability, with some preparations eliciting Toll-like receptor 2 (TLR2)-mediated immunostimulatory responses in monocytes and macrophages, while others exhibited anti-inflammatory effects. Label-free quantitative proteomics revealed that pro-inflammatory preparations contained CHO host cell proteins (HCPs). Further purification by cation exchange chromatography (CIEX) substantially removed these immunomodulatory impurities, resolving batch-to-batch variations.

**Discussion:**

These findings demonstrate that, like PSG1, fully purified PSG9 exerts anti-inflammatory activity in myeloid cells. Furthermore, process-related impurities such as HCPs can significantly confound the observed bioactivity of recombinant proteins, highlighting the critical need for rigorous impurity control during functional characterization.

## Introduction

During pregnancy, extravillous and syncytiotrophoblasts of the human placenta secrete pregnancy-specific glycoproteins (PSGs) into the maternal circulation in increasing concentrations until term (1–3). PSGs (PSG1-9 and PSG11) are believed to be essential for a successful pregnancy as reduced levels of PSG1 measured with specific antibodies or with antibodies that did not distinguish between the different family members were associated with pregnancy pathologies including preeclampsia (3, 4), spontaneous abortion (5), and fetal growth restriction (6). Our group previously demonstrated that all members of the human PSGs family have a potential immunomodulatory activity due to their ability to activate latent transforming growth factor-β (TGF-β) (7–10). Studies using PSG1 as a representative member of the family showed that the activation of TGF-β1 and TGF-β2 was mediated by direct binding to their respective latency-associated peptides (LAPs) (7, 10–12) and that PSG1 induced alternative activation of macrophages and increased the number of CD4^+^ regulatory T cells *in vitro* and *in vivo* (13, 14). Like PSG1, PSG9 was previously shown to increase regulatory T-cell frequency through the TGF-β pathway, suggesting that PSG9 may play an immunomodulatory role during pregnancy (9). However, recent reports indicated that PSG9 levels are increased in the serum of pregnant women with preeclampsia and could be used as a disease biomarker, but the role of PSG9 in this pregnancy pathology associated with inflammation remains unknown (15–17). To investigate whether PSG9 functions as an anti- or a pro-inflammatory mediator in myeloid cells, we produced recombinant PSG9 bearing an Fc tag in Chinese hamster ovary (CHO) cells.

Recombinant proteins are complex biologics manufactured in living systems, including *Escherichia coli*, yeast, and mammalian cell lines such as CHO cells (18). Importantly, the resulting drug product preparations may contain host cell proteins (HCPs), nucleic acids, lipids, and other process-related impurities in addition to the therapeutic protein of interest depending on the protein purification strategy used (18, 19). If not adequately removed, these host cell impurities can impact product quality, efficacy, and safety, including triggering innate immune activation (20–23). Indeed, prior studies demonstrated that trace levels of HCPs, such as proteases (24), phospholipase B-like-2 proteins (25, 26), CCL-2 (27), and flagellin (28), can increase the immunogenicity risk of recombinant biological products, such as monoclonal antibodies. The immunogenic potential and impact on product quality of host cell impurities have led to extensive efforts by industry to control HCP in biologics (29). Similar efforts are not usually used to control impurities in commercially available or laboratory-produced reagents utilized for non-clinical studies in a basic or preclinical research setting. Here, we describe the studies conducted to assess whether the ability of PSG9 to induce tumor necrosis factor α (TNFα) secretion in monocytes and macrophages was intrinsic to the rPSG9 protein or stemmed from contaminating impurities that deviated its immunomodulatory activity.

## Materials and methods

### Protein production and purification

PSG9-Fc samples were produced as previously described (9). PSG9 TEV-Fc differs from the previously published PSG9-Fc in that a seven-amino-acid sequence coding for a cysteine protease derived from tobacco etch virus was added between the last amino acid of PSG9 and the first amino acid of the Fc tag. All recombinant proteins containing the Fc tag were purified using HiTrap MabSelect PrismA columns in an AKTA pure chromatography system (Cytiva). Eluted fractions containing recombinant protein products were pooled, concentrated, and buffer exchanged with phosphate-buffered saline (PBS) in Amicon Ultra-10K centrifugal filter units. Purification steps such as ion exchange and gel filtration were carried out as previously reported (10). To determine the concentration of the protein samples, known concentrations of bovine serum albumin (BSA) standards were separated on 4%–20% NuPAGE Bis-Tris gels (Thermo Fisher Scientific) alongside purified protein samples at different dilutions. Gels were then stained with GelCode Blue (Thermo Fisher Scientific), and the protein samples were quantified by densitometry.

### TGF-β activation assay using reporter cells

The TGF-β1 activation assay using the MFB-F11 cells was performed as previously described (7, 30). Briefly, the latent TGF-β1 (Cat# 299-LTB-050, Bio-Techne) was incubated with the indicated proteins (PSG9-Fc or Fc control) at 37 °C for 1 h, and then 50 μL was added to the cells that were kept in serum-free Dulbecco’s modified Eagle’s medium (DMEM) for an 18-h incubation before the determination of the secreted embryonic alkaline phosphatase (SEAP) activity in the media. All treatments were performed in triplicate for each protein. The SEAP activity was measured at 600 nm in the GloMax plate reader (Promega); values obtained upon incubation of the reporter cells with serum-free DMEM were used as blanks and were subtracted from the reported values.

### Liquid chromatography–mass spectrometry analysis of PSG9 preparations

Tryptic digests of PSG9 preparations were generated by a typical in-solution digestion, using iodoacetamide for protein alkylation. Tryptic peptides were analyzed in data-dependent, positive ion mode using an orbitrap ascend mass spectrometer (Thermo Fisher Scientific) coupled to a Dionex 3000 Ultimate nanoC18LC system. Peptides were first loaded onto a nanotrap column (Thermo Fisher PepMap C18, 5 µm, 100 Å, 20 mm × 100 µm I.D.), then eluted onto a reversed-phase Easy-Spray column (Thermo Fisher PepMap C18, 3 µm, 100 Å, 15 cm × 75 µm I.D.) using a linear 120-min gradient of acetonitrile (2%–50%) containing 0.1% formic acid at a flow rate of 300 nL/min. Orbitrap resolution was set to 120K with a scan range of 375–1,500 *m/z*. ACG target was set to 400,000, and a dynamic exclusion filter was set to *n* = 1, duration = 9 s, and mass tolerance was set to ±10 ppm. Tandem mass spectra were acquired using the Ion Trap detector mode, with HCD fragmentation selected, and a fixed collision energy at 35%. ACG target was set to 10,000.

### Bottom-up proteomic analysis of PSG9 preparations

Tandem mass spectra were searched with proteome discoverer 2.4 (Thermo Fisher Scientific) using the Swiss Prot Rodentia (Rodents) database for reference with a precursor mass tolerance of 25 ppm and a fragment mass tolerance of 0.6 Da. Dynamic modifications included oxidation of methionine (M), and static modifications included carbamidomethylation of cysteine. Trypsin was set as the proteolytic enzyme, and maximum missed cleavage sites were set to 2. For label-free quantitation (LFQ), mass spectra were searched with PEAKS studio 11, using the label-free quantification module in identification-directed quantification mode. For this analysis, the UniProt proteome for *Cricetulus griseus* was annotated with the sequences of PSG9-Fc and PSG9TEV-F, and the search was performed with a mass error tolerance of 25 ppm and a fragment mass error of 0.6 Da. Retention time shift tolerance was set to auto detected, and feature intensity was set to ≥100,000. Protein filter settings were set as follows: analysis of variance (ANOVA) for significance method, use top three peptides, fold change between 2.0 and 64, has at least one peptide, and significance ≥5. Normalization was set to Use TIC (total ion current).

### Cell lines

RAW-blue™, HEK-Blue™ hTLR2, and HEK-Blue™ hTLR4 were purchased from InvivoGen. Briefly, cells were maintained and sub-cultured at 37°C in growth medium [DMEM, 4.5 g/L glucose, 2 mM L-glutamine, 10% (v/v) fetal bovine serum (FBS), penicillin–streptomycin (100 U/mL–100 µg/mL] supplemented with 1× HEK-Blue™ Selection and 100 µg/mL Normocin. THP‐1 cells were obtained from ATCC and maintained in RPMI‐1640 medium (Thermo Fisher Scientific) containing 10% heat-inactivated FBS, 1 mM sodium pyruvate, and 4.5 g/L glucose. RAW264.7 were obtained from ATCC and were cultured and maintained in DMEM (Thermo Fisher Scientific) containing 10% heat-inactivated FBS, 1% penicillin–streptomycin, and 100 μg/mL Normocin (InvivoGen).

### Animals

C57BL/6 (WT) and C57BL/6-TLR2 KO mice were obtained from The Jackson Laboratory (JAX; Bar Harbor, ME, USA) and bred and maintained under specific pathogen-free conditions in the Association for Assessment and Accreditation of Laboratory Animal Care International (AAALAC International)-accredited animal facility of the Division of Veterinary Medicine of the US Food and Drug Administration (FDA) (Silver Spring, MD, USA). All animal procedures were conducted in accordance with the recommendations outlined in the *Public Health Service Policy on Humane Care and Use of Laboratory Animals* (31).

### RAW-BLUE cells and HEK 293 h-TLR transfected cells

RAW264.7 cells carrying the SEAP reporter construct inducible by NF-κB/AP-1 activation (InvivoGen), termed RAW-BLUE cells, were plated at 100,000 cells per well in 100 µL in a flat-bottom 96-well plate. Protein samples were added to the plate and stimulated after 24 h. Then, 180 µL of Quanti-blue (InvivoGen) was added per well in a 96-well flat bottom plate with 20 µL of supernatant from the cells. After incubation at 37 °C for 2 h, the SEAP activity was measured at 600 nm with the GloMax plate reader (Promega).

HEK-BLUE-hTLR2 and HEK-BLUE-hTLR4 cells were detached using trypsin for 2–3 min at room temperature (RT). Protein samples were added to a flat-bottom 96-well plate at 20 µL/well. Positive controls LPS-B5 (from *E. coli* 055:B5, ultrapure, Cat# tlrl-pb5lps, InvivoGen) were also added at 20 µL/well. As a negative control, sterile, endotoxin-free water was used. A suspension of 280,000 cells/mL of HEK-Blue hTLR2 cells or of 140,000 cells of HEK-BLUE-hTLR4 was prepared and 180 µL of the cell suspension (~50,000 cells). The plate was incubated at 37 °C for 16 h. Then, 180 µL of Quanti-blue (InvivoGen) was added per well in a 96-well flat bottom plate with 20 µL of supernatant from the cells. After incubation at 37 °C for 2 h, the SEAP activity was measured at 600 nm with the GloMax plate reader (Promega).

To confirm the identity of the TLR that interacts with TLR2, we used anti-TLR1 Ab (Invivogen, Cat# mabg-htlr1-2), anti-TLR6 Ab (Invivogen, Cat# mabg-htlr6-2), and anti-TLR2-Ab (Invivogen, Cat#maba-htlr2-2). HEK-BLUE-hTLR2 was pre-incubated for 1 h with neutralizing anti-TLR1 Ab (1 μg/mL) or isotype control Ab before treatments with proteins PSG9 (4 μg/mL) or Fc control (4 μg/mL). PAM3C SK4(cat# tlrl-pms, InvivoGen) (0.5 ng/mL) was used as a positive control. Similarly, HEK-BLUE-hTLR2 cells were pre-incubated with anti-TLR2 and anti-TLR6 Abs for 1 h before treatment with PSG9 (4 μg/mL) or Fc (4 μg/mL) control protein. FSL-1 (Cat# tlrl-fsl, InvivoGen) (1 ng/mL) was used as positive control. The plate was incubated at 37 °C for 16 h. Then, 180 µL of Quanti-blue (InvivoGen) was added per well in a 96-well flat bottom plate with 20 µL of supernatant from the cells. After incubation at 37 °C for 2 h, the SEAP activity was measured at 600 nm with a GloMax plate reader (Promega).

### Cell treatments

A suspension of 2 × 10^5^ cells/mL of THP1 cells with 5 ng/mL of PMA was prepared and 500 μL was added to a 48-well plate. Plates were incubated at 37 °C in 5% CO_2_ for 72 h and then supernatant was removed. Protein samples were added to the well in 500 μL and supernatants were harvested 24 h later. A suspension of 1.8 × 10^6^ cells/mL of RAW 264.7 cells was prepared and 100 μL of the cell suspension was added to a 96-well plate in triplicate for 3 h. Treatments were added to the plate in 50 μL for a total volume of 150 μL, and the plate was incubated at 37 °C in 5% CO_2_ for 24 h.

Monocytes were isolated by counterflow centrifugal elutriation (by the NIDCR Combined Technical Research Core facility) from peripheral blood of healthy male donors (ages 21–50) obtained from the NIH Blood Bank (Bethesda, MD, USA). All samples were de-identified by the NIH Blood Bank. Monocytes were seeded at 3.5 to 4 × 10^5^ cells per well in macrophage serum-free media in a 96-well plate in 100 μL and treated with PSG9-Fc and PSG1-Fc for 24 h. Cell supernatants were collected and human TNFα levels were determined by enzyme-linked immunosorbent assay (ELISA) using the DuoSet ELISA kits according to the manufacturer’s instructions (Cat# DY210, R&D Systems).

To generate bone marrow-derived macrophages (BMDMs), femurs were isolated from 6- to 8-week-old C57BL/6 mice and C57BL/6-TLR2 KO mice. Bone marrow cells were flushed out of the femurs using 10 mL of RPMI medium into Petri dishes. Cells were centrifuged and resuspended in RPMI medium with M-CSF (Peprotech) added at 25 ng/mL on day 0. Petri dishes were incubated at 37 °C in 5% CO_2_, and after 3 days, the media was replaced with new M-CSF. On day 7, the cells were scraped and resuspended in fresh media with M-CSF. Cells (0.35 × 10^6^ per well) were seeded in a 96-well plate and then treated in triplicate with PSG9-Fc (20 μg/mL), LPS (625 ng/mL), Pam3CSK4 (14 ng/mL), or Fc (20 μg/mL). After 24 h, cell supernatants were collected to evaluate mTNFα levels by ELISA.

### Evaluation of cell viability

Protein samples were added to a 96-well plate in 20 µL and subsequently 1 × 10^4^ RAW 264.7 cells were added to each well. The plate was incubated at 37 °C for 48 h in a humidified, 5% CO_2_ incubator. After incubation, the CellTiter 96 Aqueous One Solution (Promega) was added to each well as recommended by the manufacturer and incubated for an additional 4 h. The absorbance at 490 nm was recorded in a GloMax plate reader (Promega).

### Statistical analysis

Statistical data analysis was carried out using PRISM software (Version 10.4.2) (GraphPad Software). Data are represented as mean ± SD of triplicate wells. Statistical analyses were performed using a two-tailed Student’s *t*-test when two treatments were compared or one-way or two-way ANOVA followed by the *post-hoc* Sidak’s test when more than two groups were compared. Differences were considered significant when *p*-value < 0.05.

## Results

### PSG9, but not PSG1, induces TNFα secretion in monocytes and macrophages

PSG1 has been reported to have anti-inflammatory function in monocytes and macrophages (13, 32–34) and dendritic cells (14). Given the high level of sequence homology between PSG1 and PSG9 and shared ability to induce T-reg conversion (9) mediated by activation of latent TGF-β (10), PSG9 was presumed to exert similar anti-inflammatory activity in myeloid cells. To test this, we generated recombinant PSG9 with an Fc tag using ExpiCHO and CHO-K1 cells and purified the protein using a protein A column followed by gel filtration chromatography. As negative control, we use the mutated Fc portion of human IgG1, which is the tag present in recombinant PSG preparations. As expected, all PSG9-Fc preparations activated latent TGF-β as indicated by the response of the MFB-F11 reporter cells, which contain TGF-β responsive SMAD-binding elements coupled to a SEAP reporter gene, as previously reported (10, 30) (**Figure 1A**). Interestingly, whereas PSG1-Fc did not induce secretion of the pro-inflammatory cytokine TNFα in murine RAW 264.7 cells, primary human monocytes, or PMA-treated THP1 cells, PSG9-Fc induced robust TNFα secretion in all three cell preparations (**Figures 1B–D****).** Since cytotoxicity and cellular stress can induce TNFα secretion (35, 36), we then tested whether PSG9-Fc affected the viability in RAW264.7 cells. We found no effects of PSG9-Fc in cell viability when compared to the protein composed of only the Fc tag (**Supplementary Figure 1**). These results suggested that, despite their close homology, PSG family members may have fundamentally distinct immunomodulatory effects, which could correspond with reports indicating that lower than normal levels of PSG1 were found in women diagnosed with preeclampsia, while elevated levels of PSG9 were detected in the maternal serum of the preeclampsia patients (3, 15–17, 37).

**Figure 1:**
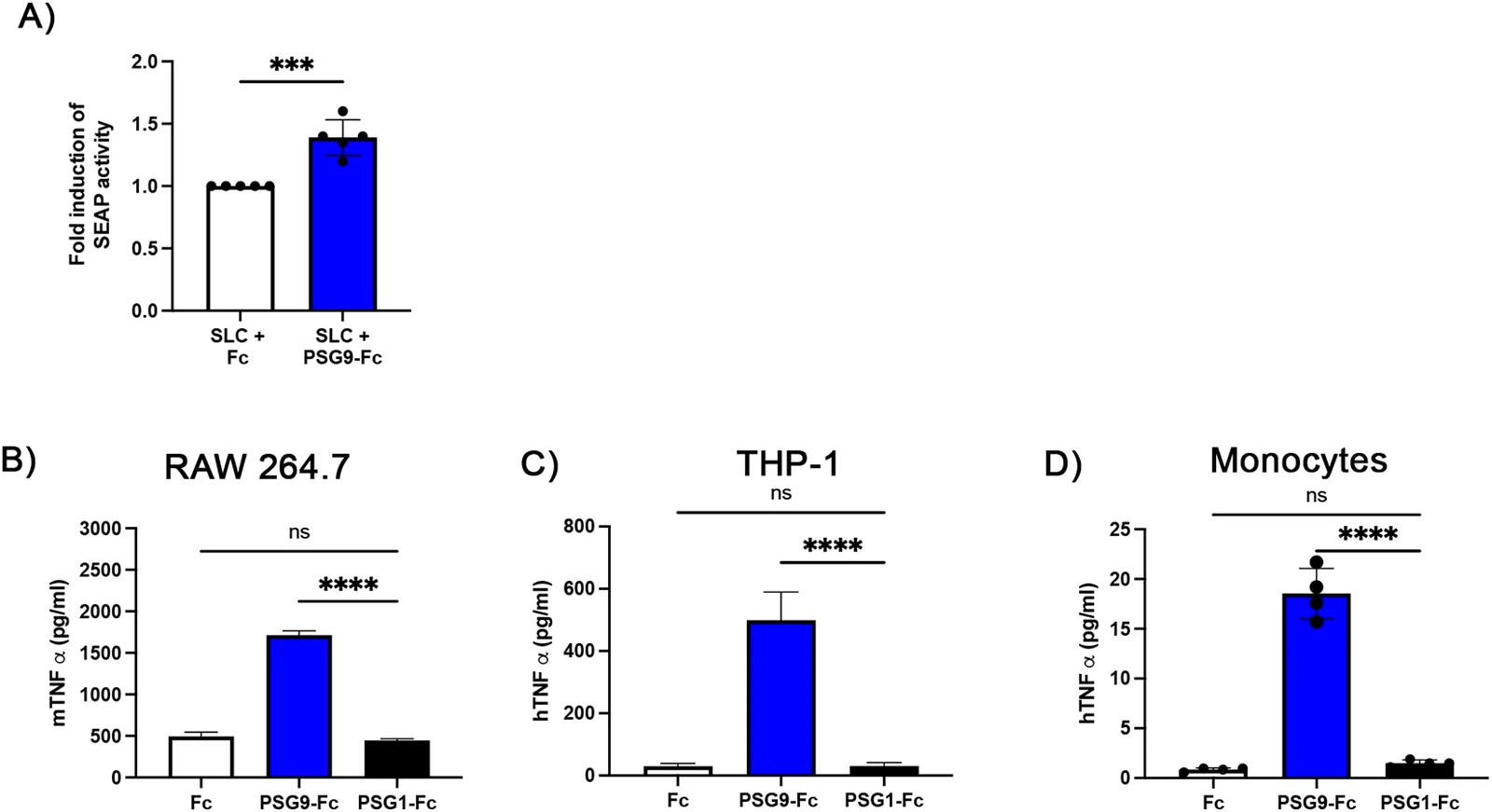
Different samples of PSG9 activate latent TGF-β and preparations of PSG9 induce secretion of TNFα in murine and human cells. **(A)** The small latent complex of TGF-β1 (SLC) was incubated with the Fc control protein (considered as 1) or different preparations of PSG9-Fc (indicated as dots representing the average of triplicate wells per protein) prior to addition to the MFB-11 reporter cells as described in the Materials and Methods section. Results are expressed as fold difference over Fc control. **(B–D)** Cells were treated with 20 mg/mL of the Fc control, PSG9-Fc, or PSG1-Fc preparations in triplicate wells for 24 h and TNFα was measured in the supernatants by ELISA. All experiments **(A–C)** were performed in triplicate wells in three independent experiments. Results **(D)** shown are representative of four donors. Results are presented as the mean ± SD and statistical significance is indicated by ***p < 0.001 and ****p < 0.0001 as determined by an unpaired *t*-test or one-way ANOVA test followed by Sidak’s post-test. The symbol ns means non significance.

### The pro-inflammatory activity of the PSG9 is mediated through TLR2

The unexpected pro-inflammatory effect of PSG9 prompted us to examine whether the protein acted by activating pattern recognition receptors (PRRs) in these cells. Because THP1 cells express TLR2 and TLR4, we first examined whether either of these receptors contributed to PSG9-induced TNFα production by using HEK293 cells that stably express either TLR2 or TLR4/CD14/MD-2. The results showed that the PSG9 domain but not the Fc domain significantly triggered NF-κB activation in HEK-TLR2 cells (**Figure 2A**) but not in HEK-TLR4 cells (**Figure 2B**). Using WT and TLR2-deficient BMDMs, we confirmed that TLR2 played a role in PSG9-induced TNFα production (**Figure 2C**). Moreover, blocking TLR2 with an anti-TLR2 antibody significantly suppressed IL-8 production in PSG9-treated human THP1 macrophages (**Figure 2D**). These findings indicated that the activity of PSG9 was mediated through TLR2, not TLR4.

**Figure 2:**
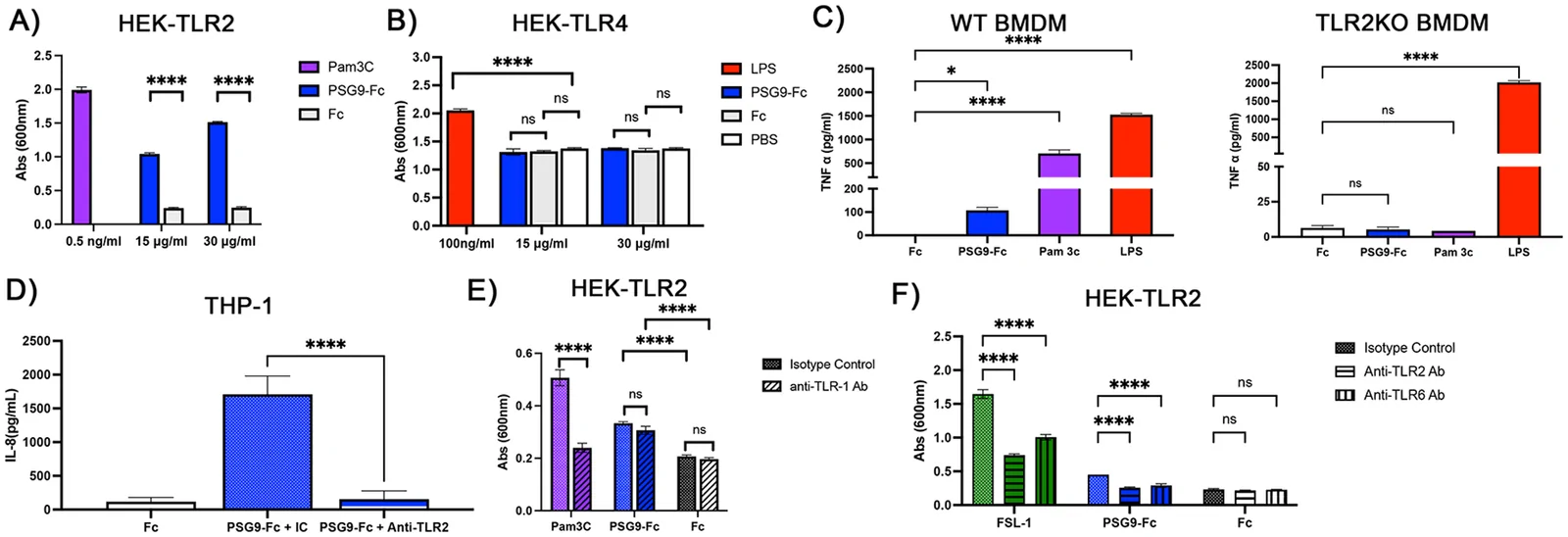
Contaminants in PSG9 preparations signal through TLR2/6. **(A)** HEK-Blue TLR2 cells were treated with increasing concentrations of pro-inflammatory PSG9 preparation (PSG9-Fc), Fc control protein, or Pam3C for 24 h. **(B)** HEK-Blue TLR4 cells were treated with increasing concentrations of pro-inflammatory PSG9 preparation (PSG9-Fc), Fc control protein, or LPS for 24 h. **(C)** Bone marrow-derived macrophages from WT and TLR2KO mice were treated with Fc, PSG9-Fc, Pam3C, and LPS for 24 h. TNFα was measured from the collected supernatants by ELISA. **(D)** IL-8 measured in supernatant of THP1 cells differentiated with PMA (5 ng/mL) for 72 h and then treated with Fc control protein, or PSG9-Fc pre-incubated with anti-TLR2 Ab or isotype control (IC). **(E)** HEK-TLR2 cells were pre-incubated for 1 h with neutralizing anti-TLR1 Ab before treatment with PSG9-Fc, Fc control protein, or Pam3C. **(F)** HEK-TLR2 cells were pre-incubated with anti-TLR2 Ab and anti-TLR6 Ab, respectively, before treatment with FSL-1, PSG9-Fc, or Fc control protein. All experiments were performed in triplicate wells in three independent experiments, and results are presented as the mean ± SD. Statistical analysis was carried out with one-way or two-way ANOVA test followed by Sidak’s post-test. Statistical significance is indicated by *p < 0.05 and ****p < 0.0001. The symbol ns means non significance.

TLR2 is expressed on the cell surface and forms heterodimers with either TLR1 or TLR6 to initiate pro-inflammatory responses (38). To assess whether TLR1, TLR6, or both were involved in the pro-inflammatory activity of PSG9, anti-TLR1 and anti-TLR6 specific antibodies were used to block receptor binding sites. While, as expected, TLR1 blocking reduced the response to the TLR2/1 agonist Pam3CSK4, the blocking did not attenuate NF-κB activation induced by PSG9 (**Figure 2E**). In contrast, blocking TLR6 suppressed NF-κB activation in response to both the TLR2/6 agonist FSL-1 and PSG9 (**Figure 2F**), indicating that the pro-inflammatory activity of PSG9 was mediated through TLR2/6, and not through TLR2/1.

### Host cell protein contamination contributes to the pro-inflammatory activity of PSG9 preparations

The unexpected TLR2/6-mediated pro-inflammatory activity raised the possibility that HCP or microbial contaminants, such as mycoplasma or other impurities introduced during protein production that were co-purified with PSG9, might be responsible for the inflammatory response (39, 40). To test whether process-related impurities contributed to the immunomodulatory activity of the PSG9 preparations, we subjected the protein to cation exchange chromatography (CIEX) after the gel filtration step to enrich the target protein and reduce potential immunostimulatory impurities. This additional purification step increased purity from ~76% and ~87% for PSG9-Fc_1_ and PSG9-Fc_2_ to ~99%, and ~98%, respectively, based on semi-quantitative label-free quantitative peak area measurements (**Figure 3**). These “polished” PSG9 batches were then tested for the ability to induce NF-κB activation in HEK-TLR2 reporter cells. As shown in **Figure 4**, purified PSG9 no longer triggered TLR2-mediated NF-κB activation, indicating that the additional purification step removed contaminants that induced TLR2 activation. Based on these results, we concluded that the pro-inflammatory activity of PSG9 was associated with the presence of trace levels of process-related contaminants such as HCP rather than to PSG9 itself, and that the intrinsic properties of PSG9 are not pro-inflammatory. Importantly, there was no difference in the pre- and post-CIEX PSG9-Fc preparations in the ability to activate latent TGF-β as indicated by the response of the MFB-F11 reporter cells (30) (**Supplementary Figure 2**).

**Figure 3:**
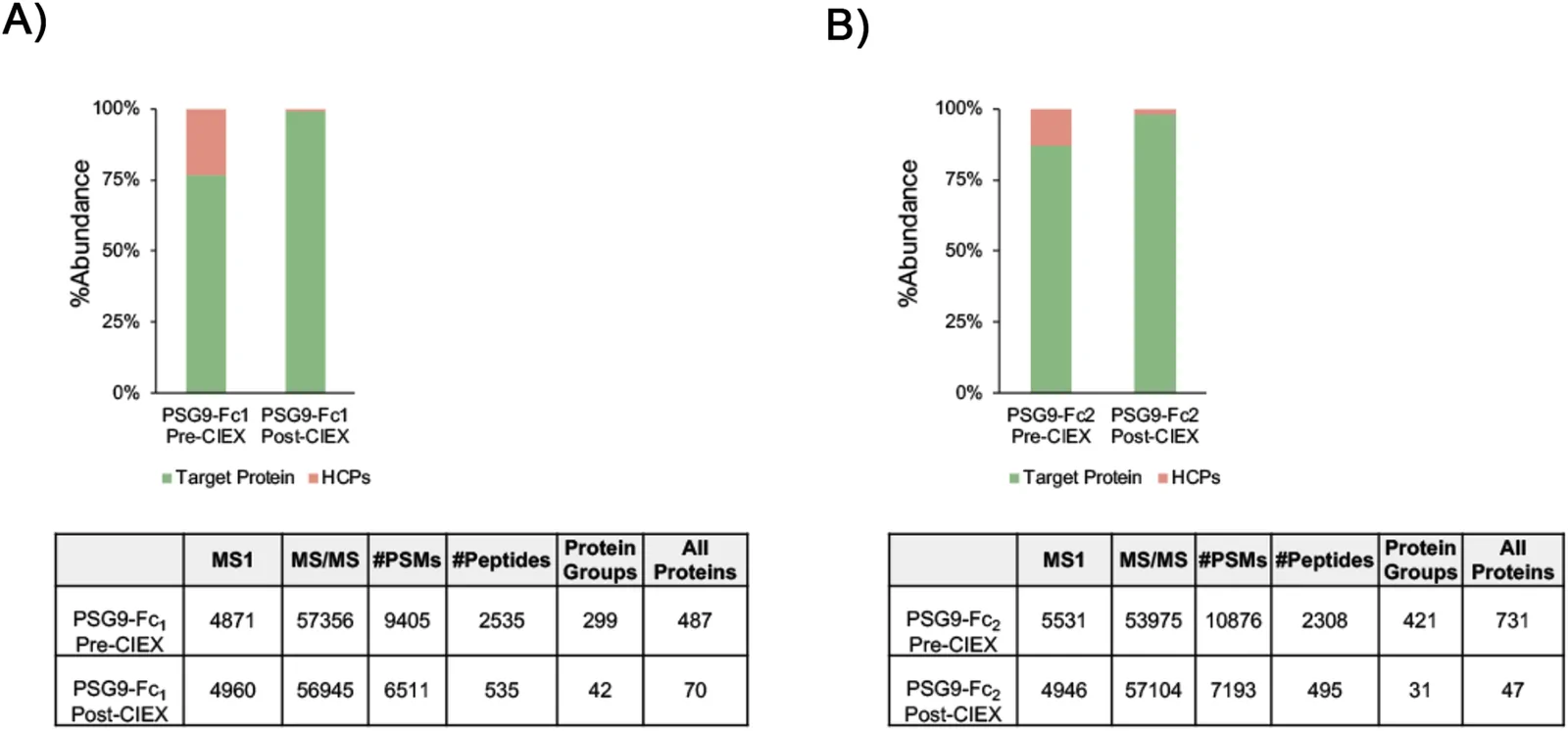
Identification of host cell proteins in pro-inflammatory PSG9 preparations by label-free quantitative proteomics (LFQ). LFQ proteomic analysis of two PSG9-Fc preparations identified contaminating host cell proteins (HCPs) that were greatly reduced after cation exchange chromatography (CIEX). **(A)** LFQ analysis of PSG9-Fc_1_ before (Pre-CIEX) and after (Post-CIEX) and **(B)** LFQ analysis of PSG9-Fc_2_ before (Pre-CIEX) and after (Post-CIEX) demonstrate a reduction of total protein identifications after CIEX. Based on semi-quantitative relative-abundance measurements. HCPs were reduced from ~23% to ~1% after CIEX in PSG9-Fc P_1_ **(A)** and from 13% to 1.8% in PSG9-Fc P_2_ **(B)**. Data in bar graphs are presented as % total abundance based on the summed peak area of all peptides identified for a given protein.

**Figure 4:**
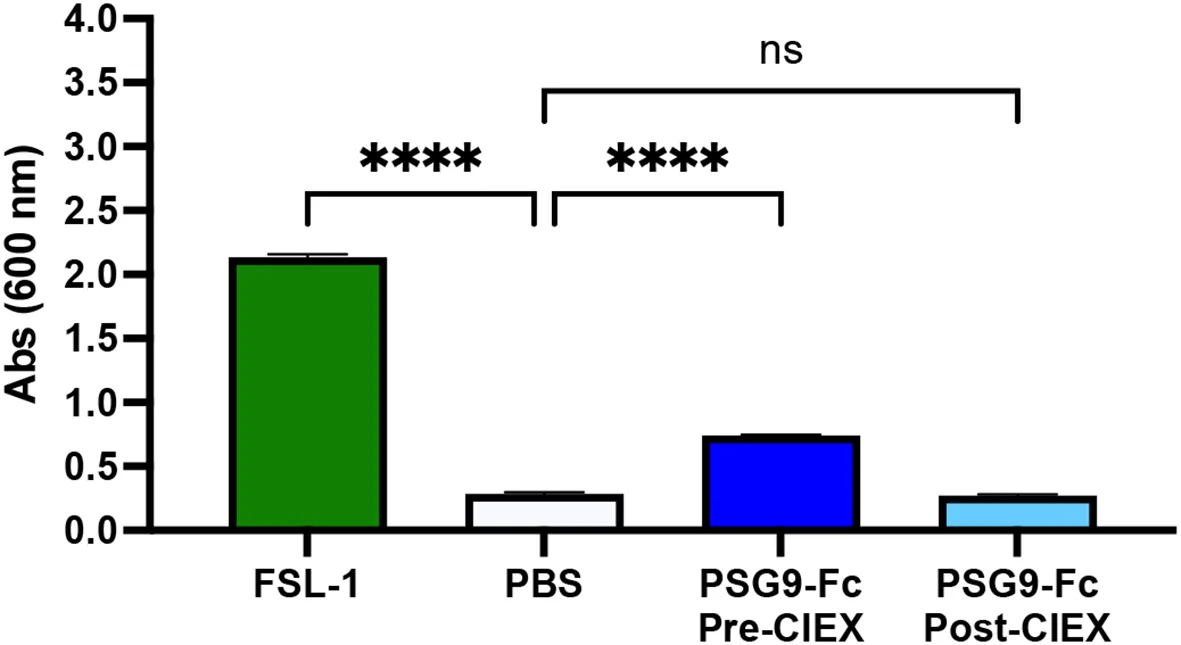
Host cell protein contamination contributes to the pro-inflammatory activity of the PSG9-Fc preparations. HEK-Blue TLR2 cells were treated with 10 µg/mL of pro-inflammatory PSG9-Fc Pre- or Post- CIEX, as indicated. FSL-1 and PBS were used as positive and negative controls, respectively. All experiments were performed in triplicate wells in three independent experiments and results are presented as the mean ± SD. Statistical significance is indicated by ****p < 0.0001. The symbol ns means non significance.

### Identification of impurities in CHO-derived PSG9

To attain a better understanding of the identity of potential impurities that could be shifting the immunomodulatory activity of our PSG9 preparations, we employed proteomics by mass spectrometry to identify potential HCPs or mycoplasma-derived proteins, and their relative abundance compared to PSG9. Label-free quantitative proteomics (LFQ) is a proteomics strategy that considers the three most quantifiable unique peptides of a protein of interest to determine differential protein abundance between a control and a treatment sample. LFQ approaches can provide semi-quantitative measurements of the relative abundance of each protein and, with appropriately calibrated fit for purpose assays, can also provide quantitative measurements. In this case, the LFQ data available are semi-quantitative and do not permit determination of whether HCPs in the preparation of PSG9-Fc are within human-use limits. LFQ proteomics was utilized to compare the differential abundance of HCPs, mycoplasma-derived proteins, and the PSG9 target protein in pre- and post-CIEX batches. No mycoplasma protein IDs were obtained using the UniProt mycoplasma database (data not shown). Importantly, the HCP characterization showed that CIEX step reduced the total number of proteins in the samples from 487 to 70 in one batch (**Figure 3A**, bottom), and from 731 to 47 proteins in the other batch (**Figure 3B**, bottom). As shown in **Table 1**, for PSG9-Fc_1_ pre-CIEX, the top 10 most abundant HCPs identified included granulins (G3HLK3), L-lactate dehydrogenase OS (G3I255), pyruvate kinase (G3H3Q1), peroxiredoxin (G3GYP9), clusterin (G3HNJ3), calreticulin (G3HCX8), endoplasmic reticulum chaperone BIP (G3I8R9), synaptic vesicle membrane protein VAT-1-like (G3HM03), L-lactate dehydrogenase A chain (G3H0C9), and lysosomal alpha-glucosidase (G3HTE5). For PSG9Fc P_2_ pre-CIEX, the 10 most abundant proteins included hemopexin (G3INL3), granulins (G3HLK3), peroxidase (G3HBI1), synaptic vesicle membrane protein VAT-1-like (G3HM03), clusterin (G3HNJ3), beta-1 metal-binding globulin (G3GZG6), laminin subunit alpha-5 (G3HGW6), pyruvate kinase (G3H3Q1), and guanine nucleotide-binding protein subunit beta-2-like 1 (G3HK00). For comparative purposes, the corresponding peak area and the LFQ ratio (Area of protein in post-CIEX batch/Area of protein in pre-CIEX batch) of these proteins are presented in **Table 1** for all batches. Of note, several HCPs identified in high abundance in pre-CIEX PSG9 batches, such as peroxiredoxin, pyruvate kinase, and clusterin, have been regarded by the biopharmaceutical industry as high-risk HCPs due to their potential impact on product quality attributes, including immunogenicity (29). In most cases, the HCPs of concern were greatly reduced by CIEX, except for peroxidase, which was reduced to a ratio of 0.03 (6.90E + 07 area pre-CIEX, 2.20E + 06 area post-CIEX) in post-CIEX PSG9-Fc2 but remained stable at a ratio of 1.12 (2.39E + 06 area pre-CIEX, 2.96 E + 06 area post-CIEX) in post-CIEX in PSG9-Fc1. While the HCPs identified in this analysis represent proteins that the biopharmaceutical industry regards as high-risk HCPs, without direct evidence of their involvement in TLR2 activation, we are only able to suggest an association between their presence in recombinant proteins and a contaminant pro-inflammatory effect.

**Table 1:** Top 10 HCPs in pro-inflammatory fractions pre-CIEX and LFQ ratio post-CIEX.

Accession	PSG9-Fc_1_ pre-CIEX	PSG9-Fc_2_ pre-CIEX	PSG9-Fc_1_ post-CIEX	PSG9-Fc_2_ post-CIEX	Avg. mass	Description
G3HQM6|G3HQM6_CRIGR	**1.31E+08**	ND	4.01E+07	ND	92,623	Endoplasmin OS = *Cricetulus griseus* OX = 10029 GN = Hsp90b1 PE = 3 SV = 1
G3GZW8|G3GZW8_CRIGR	**1.10E+07**	ND	2.72E+06	ND	20,205	Phosphoglycerate mutase 1 OS = *Cricetulus griseus* OX = 10029 GN = I79_003415 PE = 4 SV = 1
G3HH30|G3HH30_CRIGR	**7.50E+06**	ND	2.91E+06	ND	35,791	Aldo-keto reductase family 1 member B OS = *Cricetulus griseus* OX = 10029 GN = Akr1b1 PE = 3 SV = 1
G3GXB0|G3GXB0_CRIGR	**6.23E+06**	ND	3.09E+06	ND	23,423	ARHGDIA OS = *Cricetulus griseus* OX = 10029 GN = Arhgdia PE = 3 SV = 1
G3HBI1|G3HBI1_CRIGR	ND	**1.07E+09**	ND	1.04E+06	165,365	Peroxidase OS = *Cricetulus griseus* OX = 10029 GN = Pxdn PE = 4 SV = 1
G3H0U6|G3H0U6_CRIGR	ND	**8.48E+08**	ND	1.10E+06	50,052	Protein disulfide-isomerase OS = *Cricetulus griseus* OX = 10029 GN = I79_003765 PE = 3 SV = 1
G3INL3|G3INL3_CRIGR	ND	**8.43E+08**	ND	3.48E+06	51,756	Hemopexin OS = *Cricetulus griseus* OX = 10029 GN = Hpx PE = 3 SV = 1
G3HLK3|G3HLK3_CRIGR	**6.38E+07**	**5.01E+08**	2.96E+07	4.22E+07	63,924	Granulins OS = *Cricetulus griseus* OX = 10029 GN = I79_011593 PE = 3 SV = 1
G3HGW6|G3HGW6_CRIGR	ND	**3.50E+08**	ND	1.34E+07	405,897	Laminin subunit alpha-5 OS = *Cricetulus griseus* OX = 10029 GN = I79_009858 PE = 4 SV = 1
G3H6V7|G3H6V7_CRIGR	ND	**2.28E+08**	ND	3.54E+06	50,522	Lipoprotein lipase OS = *Cricetulus griseus* OX = 10029 GN = I79_006077 PE = 3 SV = 1
G3HM03|G3HM03_CRIGR	2.98E+06	**2.02E+08**	7.92E+06	6.64E+06	28,523	Synaptic vesicle membrane protein VAT-1-like OS = *Cricetulus griseus* OX = 10029 GN = I79_011747 PE = 4 SV = 1
G3HIM1|G3HIM1_CRIGR	ND	**1.95E+08**	ND	2.56E+06	334,139	Basement membrane-specific heparan sulfate proteoglycan core protein OS = *Cricetulus griseus* OX = 10029 GN = I79_010495 PE = 4 SV = 1
G3HNJ3|G3HNJ3_CRIGR	ND	**1.78E+08**	ND	5.33E+07	51,758	Clusterin OS = *Cricetulus griseus* OX = 10029 GN = Clu PE = 3 SV = 1
G3HSL4|G3HSL4_CRIGR	ND	**1.56E+08**	ND	1.62E+07	96,643	Elongation factor 2 OS = *Cricetulus griseus* OX = 10029 GN = I79_013848 PE = 3 SV = 1
G3I8R9|BIP_CRIGR	**1.75E+08**	6.30E+07	1.99E+07	2.84E+07	72,379	Endoplasmic reticulum chaperone BiP OS = *Cricetulus griseus* OX = 10029 GN = HSPA5 PE = 1 SV = 1
G3I2D3|G3I2D3_CRIGR	**1.37E+07**	1.83E+07	6.36E+06	6.31E+06	15,223	40S ribosomal protein S20 OS = *Cricetulus griseus* OX = 10029 GN = I79_017563 PE = 3 SV = 1
G3GU76|G3GU76_CRIGR	**1.14E+07**	1.43E+07	5.45E+06	1.68E+06	29,887	60S acidic ribosomal protein P0 OS = *Cricetulus griseus* OX = 10029 GN = I79_001228 PE = 3 SV = 1
G3HKG9|G3HKG9_CRIGR	**1.14E+07**	1.43E+07	5.45E+06	1.68E+06	30,606	60S acidic ribosomal protein P0 OS = *Cricetulus griseus* OX = 10029 GN = I79_011192 PE = 3 SV = 1
G3IDL7|G3IDL7_CRIGR	**3.49E+07**	1.20E+07	1.08E+07	5.69E+06	22,696	Heat shock cognate 71 kDa protein OS = *Cricetulus griseus* OX = 10029 GN = I79_021800 PE = 3 SV = 1

Bold = top 10 abundant HCP.

ND, not detected.

### Post-CIEX PSG9 has anti-inflammatory activities

After confirming the non-inflammatory property of the polished PSG9, we next assessed whether post-CIEX PSG9 would exert the expected anti-inflammatory activity previously shown for PSG1. Treatment with polished PSG9 attenuated NF-κB activation induced by LPS (**Figure 5A**) and, to a lesser extent, heat-killed *Listeria* monocytogenes (HKLM) in RAW-blue cells (**Figure 5B**). These findings indicated that PSG9 had anti-inflammatory activity, dampening the pro-inflammatory response induced by these different TLR ligands.

**Figure 5:**
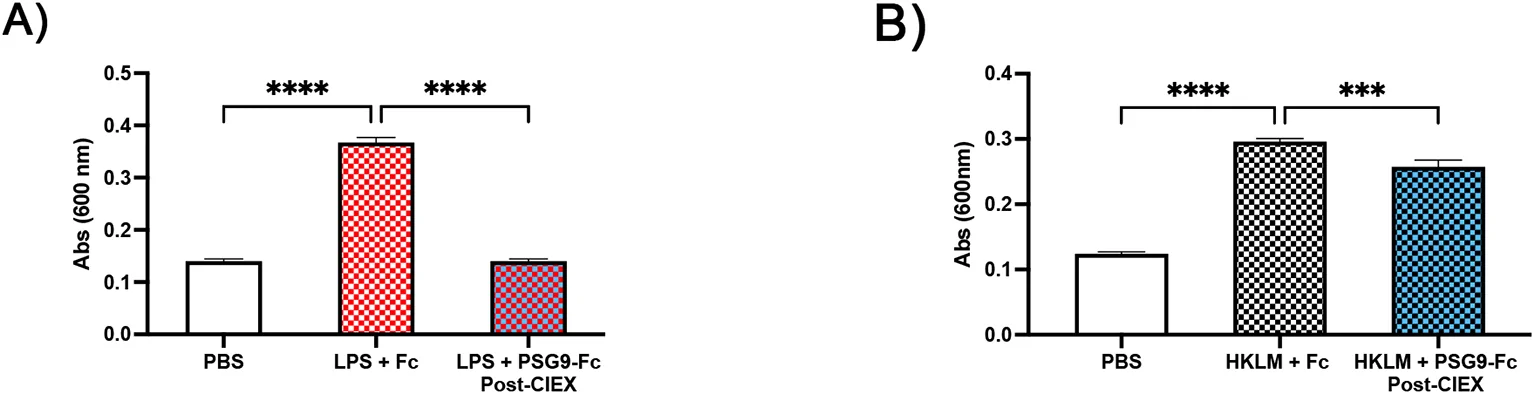
Purified PSG9 has anti-inflammatory activities as evaluated by the reduction of the NF-kB activation in response to TLR4 and TLR2 ligands. RAW-Blue cells were treated with 40 mg/mL of purified PSG9 preparation (PSG9-Fc Post-CIEX), or Fc control proteins for 24 h in the presence of LPS (1 ng/mL) **(A)** or heat-killed *Listeria* monocytogenes (1 × 10^7^ cells/mL) **(B)**. All experiments were performed in triplicate wells in three independent experiments and results are presented as the mean ± SD. Statistical significance is indicated by ***p < 0.001 and ****p < 0.0001.

## Discussion

Production of recombinant proteins has become routine over the last 50 years, which has accelerated biological discovery and drug development. In this study, we generated recombinant PSG9 to evaluate its function but unexpectedly observed that the PSG9 preparations exhibited pro-inflammatory activity in myeloid cells. Given that PSG9 consistently activated TGF-β, we questioned whether the observed pro-inflammatory activity was intrinsic to PSG9 or instead reflected process-related impurities. Additional purification by CIEX eliminated TLR2/6-mediated NF-κB activation without affecting TGF-β activation, suggesting that the pro-inflammatory activity was not an inherent property of PSG9. Proteomic analysis further revealed a substantial reduction in HCPs after polishing based on semi-quantitative LFQ measurements, supporting that trace-level contaminants could contribute to the TLR2/6-driven inflammatory responses. Together, our findings suggest that the impurities present in PSG9 preparations skewed the biological activity of PSG9 from an anti-inflammatory protein into a pro-inflammatory protein. These findings underscore the potential for HCPs and other impurities to mislead research and development of biologics.

Tagging proteins of interest along with affinity-based chromatography has improved the efficiency of protein purification (41–44). The recombinant PSG9 we generated were Fc-tagged proteins that were purified using protein A chromatography, a universal method commonly used to purify Fc fusion proteins. PSG9 preparations were further purified by gel filtration. However, additional purification by CIEX was needed to surpass 90% purity by semi-quantitative mass spectrometry analysis to avoid an unphysiological pro-inflammatory effect associated with impurities in PSG9 preparations. Of note, we routinely use the mutated Fc portion of human IgG1 as a control because it is the tag present in our recombinant PSG preparations, enabling Protein A affinity chromatography. In our studies, cells treated with the Fc control protein showed no difference compared to PBS-treated controls. Although the proteins share the amino acid composition corresponding to the Fc tag, there are considerable differences that correspond to the PSG9 sequence, and they also differ in molecular weight and carbohydrate composition. Therefore, despite following the same manufacturing protocol and using the same equipment, the co-purified HCPs and process-related impurities are not necessarily expected to be identical. Indeed, the absence of pro-inflammatory activity in the Protein A-purified Fc control confirms that its impurity profile differs from that of PSG9-Fc. This strongly suggests that the host-cell- or fermentation-derived proteins need to be separately evaluated for each product rather than rely on a manufacturing platform.

During the manufacturing workflow of recombinant proteins, HCPs are progressively secreted into the culture media (45, 46). To mitigate the risk posed by the HCP and other fermentation remnants that could copurify with the product, several methods to measure and characterize HCP impurities have been developed, including ELISAs and orthogonal analytical methods such as proteomics by mass spectrometry (47). This risk of HCP and other fermentation-associated impurities is well known for therapeutic proteins, as stated in the regulatory guidelines by the FDA and other regulatory agencies, which emphasize rigorous characterization and control of impurities. This has led to generally accepted thresholds of <100 ppm (100 ng HCPs/mg of therapeutic protein) for HCPs and <10 ng of residual DNA per dose (19, 48). While this practice is generally adopted for biopharmaceutical drugs, it does not apply to recombinant proteins produced for non-clinical research, potentially leading to misleading results.

Several endogenous ligands produced by cells during fermentation processes that signal through TLR2 and can initiate an unwanted inflammatory response have been identified, including human β-defensin-3 (49), high mobility group box 1 protein (HMGB1) (50), hyaluronan fragments (51), and heat shock proteins (52). In our study, we identified several HCPs in the pro-inflammatory preparations that have been regarded by the pharmaceutical industry as potentially high-risk HCPs, including peroxiredoxin, clusterin, and calreticulin (29). Treatment of monocyte-derived dendritic cells (MoDCs) with peroxiredoxin-1 (PRDX1) increased the expression of maturation markers such as CD86, suggesting that PRDX1 may possess intrinsic adjuvant potential and contribute to innate immune activation (53). Peroxiredoxin has also been reported to promote inflammatory responses through TLR2 and TLR4 stimulation (54–56). Similarly, Ke et al. showed that secreted clusterin potentially binds to TLR2 and that induced macrophages express TNF-α (57). Clusterin has also been shown to promote macrophage recruitment by directly stimulating macrophage chemotaxis and inducing the expression of chemotactic cytokines, including TNF-α (58). Finally, calreticulin, derived from mucus proteins, activated neutrophils by interacting with TLR2 and TLR4 receptors (59). These suggest that those HCPs could be involved in the observed TLR-2-mediated immunomodulatory effect of the PSG9 preparations. Importantly, it was not the aim of our study to identify the specific impurities that could cause the pro-inflammatory profile, and therefore, our study did not directly assess whether these specific HCPs were associated with TLR2 activation or there were other impurities that were also cleared by CIEX purification that led to the loss of TLR2 activity. Although we did not identify which HCPs were responsible for modulating changes in the function of PSG9, we provided a list in **Table 1** of potential HCP candidates that could induce innate immunogen activation and were reduced by CIEX.

Mycoplasma is a common and often undetected contaminant of cells in culture, and mycoplasma-derived lipoproteins are known to activate innate immune responses via TLR2 signaling (39). However, searching against the mollicutes, proteome databases indicated that none of the PSG9 preparations contained detectable mycoplasma-related proteins (data not shown).

In this study, we performed bottom-up label-free quantitative proteomics as an unbiased method to profile. The major advantage of this mass spectrometry-based approach for HCP profiling was this approach can identify low-abundant HCPs with as little as two unique peptides, and without the limitations of antibody specificity, which is a common restriction of ELISA-based HCP profiling. Label-free quantitative proteomics can provide semi-quantitative measurements of differential protein abundances in complex proteomic samples through the consideration of several quantifiable unique peptides of a given protein; however, the currently available LFQ data do not permit calibrated quantitative HCP measurements to determine if the HCP content is within human-use limits. Additionally, quantitative HCP analysis by ELISA was not performed, primarily because the PSG9-Fc fusion proteins were produced for structural and functional and immunological characterization purposes, and not in the context of process validation for manufacturing a drug product. A validated fit-for-purpose quantitative HCP assay is required for use as a control strategy in the context of drug product manufacturing. Through this approach, we demonstrated the reduction of high-risk HCPs after CIEX, which, in turn, showed that both PSG1 and PSG9 have anti-inflammatory activity.

In summary, when we observed that recombinant PSG9 preparations unexpectedly induced TNFα secretion in monocytes and macrophages, indicating that PSG9 may have a pro-inflammatory rather than an anti-inflammatory effect in innate immune cells, we investigated whether this was an intrinsic property of PSG9. Our investigation revealed that this immunostimulatory effect was consistent with a contribution from host-cell-derived impurities that activated TLR2/6, thereby altering the activity profile of the protein of interest. Our study highlights the importance of applying quality control tests to reduce the potential contaminants present in non-pharmaceutical grade recombinant proteins, as subtle differences in culture conditions and cellular stress can result in substantially different biological properties. We hope to raise awareness on the importance of detecting these unwanted potential contaminants in non-pharmaceutical recombinant proteins by employing different detection assays including the innate immune response modulating impurities assay as a method for screening of trace levels of impurities that acting individually or in concert can induce immune activation (28, 60, 61).

## Data Availability

The mass spectrometry proteomics data have been deposited to the ProteomeXchange Consortium via the PRIDE (62) partner repository with the dataset identifier PXD083658.

## Supplementary material

Supplementary Figure 1 PSG9 does not affect cell viability. RAW 264.7 cells were treated with PSG9-Fc (40 μg/mL) and the Fc control protein (40 μg/mL). Cell viability was determined using the MTS assay. All experiments were performed in triplicate wells in three independent experiments and results are presented as the mean ± SD.

Supplementary Figure 2 Activation of the small latent complex (SLC) of TGF-β1 by representative forms of PSG9-Fc. The Fc control protein or the PSG9-Fc (post-CIEX) and PSG9-Fc (pre-CIEX) preparations were incubated with the small latent complex of TGF-β1 (SLC) before addition to the TGF-β MFB-11 reporter cells. All treatments were performed in triplicate in three independent experiments. The results of a representative experiment using one of each PSG9-Fc preparation are shown. All experiments were performed in triplicate wells in three independent experiments and results are presented as the mean ± SD. Significant differences between treatments were determined using one-way ANOVA.
